# Utility of *p63* and *p40* in Distinguishing Polymorphous Adenocarcinoma and Adenoid Cystic Carcinoma

**DOI:** 10.31557/APJCP.2019.20.10.2917

**Published:** 2019

**Authors:** Aribah Atiq, Sajid Mushtaq, Usman Hassan, Asif Loya, Mudassir Hussain, Noreen Akhter

**Affiliations:** *Department of Pathology, Shaukat Khanum Cancer Hospital, Johar Town, Lahore, Pakistan. *

**Keywords:** Adenoid cystic carcinoma, polymorphous adenocarcinoma, p63, p40

## Abstract

**Objective:**

Adenoid cystic carcinoma and polymorphous adenocarcinoma are primarily the tumor of minor salivary glands. Both show certain morphological similarities, which limit their proper diagnosis in settings where there are obscuring factors and limited biopsy material. However, there is a considerable difference in treatment and prognosis, which raises the need to distinguish these two entities. In this study, we discuss the utility of two immunohistochemical stains,* p63* and* p40*, in different combinations for distinguishing polymorphous adenocarcinoma from adenoid cystic carcinoma.

**Materials and Methods:**

Two immunohistochemical stains,* p63* and* p40*, were performed on 47 cases of adenoid cystic carcinoma and 23 cases of polymorphous adenocarcinoma.

**Results:**

36 out of 47 cases of adenoid cystic carcinoma showed* p63+ve/p40+ve* immunoprofile, followed by *p63-ve/p40-ve* immunoprofile, which is seen in10 cases of adenoid cystic carcinoma. However, 22 out of 23 cases of polymorphous adenocarcinoma displayed *p63+ve/p40-ve* immunoprofile. *p63-ve/p40+ve* is the least frequent observed immunoprofile, which is seen in only one case of adenoid cystic carcinoma.

**Conclusion:**

On combining all possible immunoprofile combinations, *p63+ve/p40-ve* immunoprofile appears to be the most sensitive profile for distinguishing polymorphous adenocarcinoma from adenoid cystic carcinoma.

## Introduction

Adenoid cystic carcinoma is one of the most common salivary gland malignancies, predominantly involving the minor salivary glands (Kokemueller et al, 2004; Dodd and Slevin, 2006). Due to wide morphological spectrum of adenoid cystic carcinoma, it has numerous differential diagnosis, most common being polymorphous adenocarcinoma, previously known as polymorphous low-grade adenocarcinoma. It is imperative to reach an accurate diagnosis, as there is difference in the prognosis, treatment and follow up of these two entities. However, distinction between adenoid cystic carcinoma and polymorphous adenocarcinoma remains a diagnostic challenge especially in small biopsies, sometimes requiring a battery of immunohistochemical stains for confirmation of diagnosis (Darling et al., 2002).

Adenoid cystic carcinoma usually arises in the fifth and sixth decades of life with no gender predilection and has an unrelenting course of disease with multiple recurrences. Histologically, the tumor has biphasic appearance, composed of basaloid appearing cells with angulated, hyperchromatic nuclei arranged in tubular, cribriform or solid patterns (Jaso and Malhotra, 2011).

In comparison, polymorphous adenocarcinoma, is exclusively confined to the minor salivary glands and has a good prognosis (Aberle et al., 1985). Histologically, they show invasive growth, multiple architectural patterns with cytological uniformity. The most common patterns are solid nests with areas showing cribriform, trabecular and papillary patterns (Paleri et al., 2008).

Adenoid cystic carcinoma and Polymorphous adenocarcinoma have overlapping histopathological features sharing common architectural patterns with contrasting nuclear features. However, assessment of these features, for diagnostic proposes can be daunting, especially in small biopsies (Turk and Wenig, 2014). Previously, many immunohistochemical markers have been investigated to differentiate between these two tumors mainly CD117, S100, c-Myb and galectin-3. However, the application and importance of these markers was never clearly established for diagnostic purposes.

Adenoid cystic carcinoma harbors a myoepithelial component. The presence of myoepithelial cells in polymorphous adenocarcinoma is debatable as it shows variable results with different myoepithelial markers.* p63* is located on chromosome 3q27-29, is a marker of myoepithelial cells. Diffuse positivity of* p63* stain is generally seen in both adenoid cystic carcinoma and polymorphous adenocarcinoma. Another antibody,* p40*, isotype of* p63* has been shown to be a more specific marker for basal and myoepithelial differentiation.* p40* is usually negative in polymorphous adenocarcinoma, whereas positive staining pattern has been observed in adenoid cystic carcinoma (Rooper et al., 2015). 

This differential pattern of staining of *p63/p40 *has been found useful in distinction between salivary gland tumor with overlapping morphological features predominantly with epithelial myoepithelial components. This study is based on study of differential patterns of* p63* and *p40* in adenoid cystic carcinoma and polymorphous adenocarcinoma.

## Materials And Methods

This was a descriptive, cross sectional study. It was carried out in Shaukat Khanum Memorial Cancer Hospital and Research Center (SKMCH and RC). Approval was obtained from the Hospital ethical committee. Forty-seven cases of adenoid cystic carcinoma and twenty-three cases of polymorphous adenocarcinoma were retrieved from the archives of department of surgical pathology of SKMCH and RC. These cases were previously diagnosed and further reviewed by two pathologists with special interest in Head and Neck pathology. No such case was included in the study in which there is diagnostic uncertainity. All cases were formalin fixed (10% neutral buffered formalin) and embedded in paraffin. 5-µm sections were cut from these blocks.

Two anti-bodies,* p63* and *p40*, were used in this study. The clone used for* p63* was anti-p63 (4A4) mouse monoclonal primary antibody. Concentrated and pre-diluted polyclonal antibody was used for* p40*. The normal control used for* p63* was normal prostatic tissue and for* p40*, squamous cell carcinoma. Five-micron tissue sections were cut to perform the immunohistochemistry. The buffer used for antigen retrieval was EDTA; pH 8.0, and incubation for primary antibody was done for 32 minutes. Staining was done on an automated stainer (Ventana benchmark XT for* p63* and bond III for* p40*) using standard techniques i.e, biodin-peroxidase conjugated avidin method. 

Two pathologists, blinded to the final diagnosis, interpreted the results of* p63* and *p40* independently. Nuclear positivity in ≥5% cells was considered as positive results for both antibodies in our study. Results of* p63* and* p40* stain were noted in all cases of adenoid cystic carcinoma and polymorphous adenocarcinoma. The result were recorded and compiled in four possible combination/patterns based on their relative positivity and negativity of* p63* and *p40* in all cases. The four immunohistochemical patterns were 

1-* p63+/p40* + ( IHC pattern 1)

2-* p63 +/p40* - (IHC pattern 2)

3- *p63-/p40* + (IHC pattern 3)

4- *p63- /p40* - (IHC pattern 4)

## Results

*Clinical and morphological features*

The mean age of our study population is 44.8±14.647 years, which included 39 male patients (55.7%) and 31 female patients (44.3%). The mean tumor size is 2.8971 ± 2.0233cm. Cribriform architectural pattern n=59 (84.3%) is the most frequent followed by tubular n=49(70%), papillary n=18 (25.7%) and solid n=16 (22.9%) in both the tumors. Clinical and morphological data is stratified in Table 1.

*Immunohistochemical features*

The commonest immunohistochemical pattern in 36/47 cases of adenoid cystic carcinoma is *p63+/p40+ *(76%) followed by *p63-/p40-* in 10/47(21%). Only one case out of 47 showed* p63-/p40+* staining pattern, whereas none of the cases of adenoid cystic carcinoma showed *p63+/p40- *staining pattern.

The most frequent pattern in Polymorphous adenocarcinoma is *p63+/p40-* seen in 22/23 cases (96%) with only one case showing *p63-/p40- *pattern (4%). Immunohistochemical patterns *p63+/p40+* and* p63-/p40+* are not seen in polymorphous adenocarcinoma. These results are summarized in Table 2 and 3.

**Figure 1:**
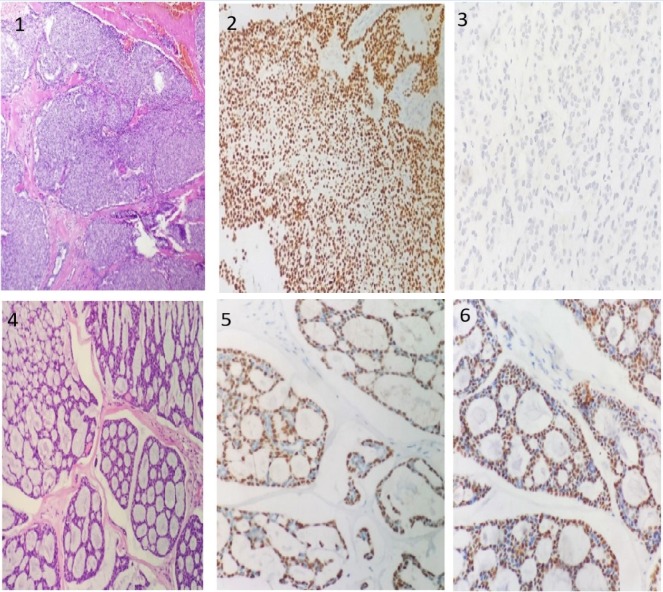
1, H &E section of polymorphous adenocarcinoma showing predominantly lobulated growth pattern. Cells are showing optically clear nuclei with clear cytoplasm;

2, Cells of polymorphous adenocarcinoma showing strong staining with p63 immunohistochemical stain;

3, polymorphous adenocarcinoma cells showing complete negative staining with p40 immunohistochemical stain;

4, H &E section of adenoid cystic carcinoma with uncommon cribriform architecture;

5, Myoepithelial cells in adenoid cystic carcinoma staining positive with p63 immunohistochemical stain;

6, strong expression of myoepithelial cells of adenoid cystic carcinoma with p40.

**Table 1:** Clinical and Morphological Features of Adenoid Cystic Carcinoma and Polymorphous Adenocarcinoma

	Mean age (S.D) years	Mean size (cm)	Gender		Margin status			Common patterns*			
			Male	Female	Involved	Uninvolved	Not assessable	Cribriform	Tubular	Solid	Papillary
Adenoid cystic carcinoma (n=47)	41.30±13.49	3.01±2.30	30 (63.8%)	17 (36.2%)	29 (61.7%)	0 (0%)	18 (38.3%)	43 (91.4%)	33 (70.2%)	7 (14.9%)	6 (12.7%)
Polymorphous adenocarcinoma (n=23)	51.96±14.2	2.66±1.3	9 (39.1%)	14 (60.9%)	12 (52.2%)	3 (13.04%)	8 (34.8%)	16 (69.6%)	16 (69.6%)	9 (39.13%)	12 (52.17%)

*More than one pattern were observed in most tumors

**Table 2:** Summary of Results of Immunohistochemical Stains* p63* and *p40*

	p63		p40	
	Positive	Negative	Positive	Negative
Adenoid cystic carcinoma	36 (76.5%)	11 (23.5%)	37 (78.7%)	10 (21.3%)
polymorphous adenocarcinoma	22 (95.6%)	1 (4.4%)	0 (0%)	23 (100%)

**Table 3:** Comparison of All Possible Immunoprofiles Utilizing* p63* and *p40*

		Diagnosis		
		Adenoid cystic carcinoma	Polymorphous adenocarcinoma	
Combination 1	p63+ve/p40+ve	36 (76%)	0	36
Combination 2	p63+ve/p40-ve	0	22 (96%)	22
Combination 3	p63-ve/p40+ve	1 (3%)	0	1
Combination 4	p63-ve/p40-ve	10 (21%)	1 (4%)	10
		47	23	70

*Comparison of all possible immunoprofiles*

Immunohistochemical pattern *p63+/p40+* (Combination 1) is seen in 36 out of 47 cases of adenoid cystic carcinoma and zero cases of polymorphous adenocarcinoma. Whereas, *p63+/p40- *staining pattern (combination 2) is seen in 22 out of 23 cases of polymorphous adenocarcinoma and zero cases of adenoid cystic carcinoma. P-value for the immunohistochemical patterns (Combination 1 and 2) in adenoid cystic carcinoma and polymorphous adenocarcinoma is p= 0.000. Thus, *p63+ve/p40+ve* profile appears to be most sensitive profile observed in adenoid cystic carcinoma and *p63+ve/p40-ve* profile appears to be most sensitive profile for polymorphous adenocarcinoma.

On comparing the results of combination *2 *(*p63+/p40-*) and *4 (p63-/p40-)* in both tumors, by applying Fisher’s extract test, p value is less than 0.0001. 

Hence, p value of immunohistochemical pattern* p63+/p40- *(Combination 2) seen primarily in polymorphous adenocarcinoma is very significant as compared two common patterns i.e.* p63+ve/p40+ve* profile and *p63-ve/p40-ve* most frequently observed in adenoid cystic carcinoma.

Combination 3 (*p63* *negative/p40* positive) was the least common pattern seen in only one case of adenoid cystic carcinoma and zero cases of polymorphous adenocarcinoma.

## Discussion

Adenoid cystic carcinoma and polymorphous adenocarcinoma are amongst the most common salivary gland tumors. 

Both tumors show contrasting nuclear features, as in adenoid cystic carcinoma there is small bland cells with angulated nuclei and compact cytoplasm. Whereas, polymorphous adenocarcinoma shows bland round cells with optically clear nuclei. Both tumors share multiple overlapping architectural patterns. However, in smaller biopsies these tumors do pose a diagnostic challenge due to multiple factors like crushing artifact, limited biopsy material, fixation artifact etc. these factors make the correct diagnosis of polymorphous adenocarcinoma possible in as few as 22 – 43 % of cases (Pogodzinski et al., 2006). 

Cribriform architectural pattern was seen in 91 % (n= 43 out of 47) and 69 % (16 out of 23) of adenoid cystic and polymorphous adenocarcinoma respectively, therefore becoming the most common, overlapping patterns in both the tumors.

Although there are numerous histological similarities, the prognosis and treatment is entirely different. The 5-year survival rate of polymorphous adenocarcinoma is 80% and that of adenoid cystic carcinoma is 64% (Schwarz et al., 2011). The treatment options are also different, as sometimes adenoid cystic carcinoma requires radical neck dissection, which is usually not indicated in polymorphous adenocarcinoma. Also, adjuvant radiotherapy is usually offered in adenoid cystic carcinoma, but not in case of polymorphous adenocarcinoma. A different prognostic outcome and management makes the distinction between the two entities and accurate diagnosis more important, both on resection specimen and small biopsies.

Previously, many antibodies i.e., S100, SMA, CD117, bcl 2 and c-myb etc. have been studied for distinction of adenoid cystic carcinoma from polymorphous adenocarcinoma (Epivantianos et al., 2007; Vargas et al., 1997; Prasad et al, 2008). In a study by Penner et al., (2002), he explained the diffuse positivity of c-kit in adenoid cystic carcinoma and loss of expression in polymorphous adenocarcinoma. However, in another study conducted by Edwards et al., (2003), they studied the c-kit expression in 49 salivary gland neoplasms (17 polymorphous adenocarcinoma, 17 monomorphous adenomas and 15 adenoid cystic carcinomas), and explained that c-kit did not appear to be helpful marker in distinguishing between adenoid cystic carcinoma from polymorphous adenocarcinoma. Likewise, *bcl-2* immunohistochemical expression was also studied by investigators to distinguish adenoid cystic carcinoma from polymorphous adenocarcinoma, but the results were not statistically significant (Meer et al., 2011). Therefore, the diagnostic utility of any of the above mentioned immunomarkers was never clearly established.

We evaluated the expression of* p63* and *p40* in both adenoid cystic and polymorphous adenocarcinoma. Their relative expression in both tumors was noted in all the possible combinations/patterns. In our results, *p63+ve/p40+ve* (76%) and *p63-ve/p40-ve* (21%) were most frequent combinations observed in adenoid cystic carcinoma. In a study conducted by Rooper et al., (2015), again the two common patterns were *p63+ve/p40+ve* and* p63-ve/p40-ve* with frequencies of 89% and 10% respectively. In our 47 cases of adenoid cystic carcinoma, there is no single case with *p63+ve/p40–ve* expression. However, there is a single cases of *p63+ve/p40–ve *expression in Bishop et al., (2014) study and this may be due to higher number of adenoid cystic carcinoma (n=101) cases in their study. However, the relative frequencies of these common patterns in adenoid cystic carcinoma are almost comparable in our studies.

The commonest pattern seen in polymorphous adenocarcinoma was *p63+ve/p40-ve* (96%) followed by one case of *p63-ve/p40-ve* in our study. This percentage is again comparable to Bishop et al study where 100% cases of polymorphous adenocarcinoma (n=11) showed *p63+ve/p40-ve* pattern.

These staining patterns, among adenoid cystic carcinoma, both *p63* and *p40* demonstrated a peripheral (abluminal) staining pattern i.e., staining in myoepthelial cell but not in ductal cells. However, different staining pattern were observed in cases demonstrating solid pattern, necrosis and increased mitotic activity. In polymorphous adenocarcinoma,* p63* demonstrated diffuse staining pattern and all cases were negative for* p40*. The different staining patterns, observed in adenoid cystic and polymorphous adenocarcinoma are shown in Figure (1-6).

On a retrospective analysis and comparison of immunoprofiles with architecture, it is seen that those cases of adenoid cystic carcinoma and polymorphous adenocarcinoma which showed *p63-ve/p40-ve* profile have high grade morphology and predominant solid pattern. In another study by Du et al., (2016), they studied myoepithelial differentiation in 109 adenoid cystic carcinomas including 38 cribriform, 35 solid and 36 tubular subtypes. They concluded that solid pattern of adenoid cystic carcinoma showed loss of myoepithelial differentiation and are associated with more aggressive behavior and poor prognosis. They also show a high proliferation index. In our study, we encountered with the same fact that all 10 cases of adenoid cystic with predominant solid pattern showed *p63-ve/p40-ve* immunoprofile with a high ki76 index. Indeed, one case of polymorphous adenocarcinoma showed *p63-ve/p40-ve *immunoprofile and that case also showed solid pattern with necrosis and increased mitotic activity. 

In summary, p values of combination pattern 2 (p63+ve/p40-ve) primarily seen in polymorphous adenocarcinoma is very significant as compared to combination pattern 1(p=0.001) and combination pattern 4 (p=000.0), mostly seen in adenoid cystic carcinoma. This shows that the combination pattern 2 which is* p63* positive and p40 negative is statistically significant pattern in polymorphous adenocarcinoma. Although diagnostic utility still requires larger studies, but it can be inferred from our study that the expression of combination number 2 in a tumor makes the possibility of adenoid cystic carcinoma highly unlikely and can be of aid in excluding this diagnostic challenge 
